# In Silico and Wet Analysis of *BAX* Gene G-248A Polymorphism and mRNA Expression in Peptic Ulcer Disease and Gastric Cancer

**DOI:** 10.3390/cimb47121005

**Published:** 2025-11-29

**Authors:** Marta Żebrowska-Nawrocka, Rafał Świechowski, Dagmara Szmajda-Krygier, Bartosz Lenda, Marek Mirowski, Michał Czaplij, Mariusz Balcerczak, Ewa Balcerczak

**Affiliations:** 1 Department of Pharmaceutical Biochemistry and Molecular Diagnostics, Medical University of Lodz, 90-151 Lodz, Poland; marta.zebrowska@umed.lodz.pl (M.Ż.-N.); dagmara.szmajda@umed.lodz.pl (D.S.-K.); bartosz.lenda@umed.lodz.pl (B.L.); ewa.balcerczak@umed.lodz.pl (E.B.); 2 Department of Surgery, District Hospital in Leczyca,99-100 Leczyca, Poland; mczaplij@gmail.com (M.C.); mariusz.balcerczak@poczta.onet.pl (M.B.)

**Keywords:** carcinogenesis, apoptosis, expression, polymorphisms, BAX, SNP G-248A, rs4645878

## Abstract

Peptic ulcer disease and gastric cancer are influenced by both environmental factors and genetic background. One such genetic factor is changes in the *BAX* gene, where G-248A decreases the activity of the *BAX* gene promoter, thereby inhibiting apoptosis and promoting carcinogenesis. The relationship between the *BAX* gene and the risk of developing these diseases has not been fully elucidated. In this study, genotyping G-248A was performed by restriction fragment-length polymorphism, and real-time PCR was employed to quantify *BAX* mRNA expression. An in silico analysis was performed using publicly available databases. The findings reveal a significant prevalence of the AA genotype in the gastric cancer group compared to healthy individuals, suggesting a potential genetic predisposition to malignancy. When peptic ulcer group and healthy controls were compared, no significant association was found. Further, in silico analyses demonstrated elevated *BAX* expression in gastric cancer tissues, correlating with advanced histological grades and improved overall survival rates. Elevated *BAX* expression, however, is associated with gastric cancer onset and could be a promising prognostic indicator. This study underlines the complex correlation between genetic factors and disease, highlighting the potential of the *BAX* gene as a biomarker for gastric cancer prognosis and therapeutic targeting.

## 1. Introduction

Peptic ulcer disease (PUD) is common condition associated with several serious complications, including bleeding, perforation, or gastric cancer. The main risk factors for the development of PUD include *Helicobacter pylori* infection and the use of non-steroidal anti-inflammatory drugs (NSAIDs). As not all patients infected with this bacterium or those taking NSAIDs have peptic ulcer disease, it has been proposed that the genetic background of the patient may also play a significant role [1,2,3]. The formation of a gastric ulcer due to damage to the gastric mucosa may be linked to impaired apoptosis. Disturbances in programmed cell death are also associated with increased cancer development. One of the genes involved in the apoptosis process is BAX, which plays a crucial role in maintaining this physiological mechanism. B-cell lymphoma 2-associated X protein (BAX), encoded by the *BAX* gene located on chromosome 19, promotes programmed cell death. Its function is regulated by TP53, and some studies show that it may also be regulated by NFkB. NFkB may reduce *BAX* gene promoter transcription, thus decreasing its activity [4,5,6]. Indeed, single-nucleotide polymorphisms (SNPs) in the promoter or the coding part of the *BAX* gene have been found to change the expression or function of the encoded protein [7,8]. One such SNP, characterized by the substitution of guanine for adenine at position 248 (rs4645878) of the promoter region of the *BAX* gene [9], may be associated with several diseases, including various types of cancer [10]. Some research suggests that the occurrence of the G-248A SNP may decrease the activity of the *BAX* gene promoter, leading to a reduced level of the pro-apoptotic *BAX* transcript, thereby inhibiting apoptosis and promoting carcinogenesis [11].

In silico studies by Wang et al. showed that the *BAX* gene could serve as a pan-tumor predictive biomarker and may function as an oncogene. The authors indicate that its increased expression was present in various cancers and was associated with proliferation, metastasis, and methylation, positively correlated with most infiltrating immune cells, and also demonstrated clinical significance, including treatment response [12].

The primary objective of this investigation is to elucidate the potential correlation between the G-248A SNP in the *BAX* gene and its mRNA expression levels, with a focus on their collective impact on susceptibility to *Helicobacter pylori* infection and the subsequent risk of developing peptic ulcer disease and gastric cancer. Additionally, the influence of *BAX* gene interactions with other genes, e.g., NFKB2 or *TP53*, on the risk of developing cancer will also be compared. The uncovered novel genetic markers may contribute to the pathogenesis of these conditions, offering insights into the molecular mechanisms driving disease progression. By integrating genetic analysis with clinical outcomes, the potential clinical relevance of these findings will also be evaluated.

## 2. Materials and Methods

### 2.1. Study Cohort

Material was collected from several independent groups of patients. The first group consisted of patients with PUD diagnosed in Department of Surgery, District Hospital, Leczyca. In all investigated cases, a gastric mucosa specimen was taken from the antrum of the stomach during routine gastroscopy. Unfortunately, the fragment of the mucosa was not sufficient for the simultaneous isolation of both nucleic acids from the same patient. Therefore, two independent patient subgroups were created: one was used for the collection of 183 DNA samples (117 women, 66 men; mean age 52 years) and the other for 36 RNA samples (21 women, 15 men; mean age 54 years).

The second group consisted of 13 patients diagnosed with gastric adenocarcinoma (5 women, 8 men; mean age 66 years) at the Department of Pathology, Medical University of Lodz, Poland, based on histopathological examination. In the patients with gastric cancer (GC), clinical stage, TNM, and histological malignancy grade were rated (see Supplemental Table S3). Tissue specimens of GC were taken during the operation and immediately frozen in liquid nitrogen. Fortunately, the amount of collected material allowed for the simultaneous isolation of both DNA and RNA from the same tissue fragment. In addition, in six GC patients, morphologically normal tissue was taken beyond the tumor margin.

The control group consisted of 86 blood samples collected from the local blood bank. The donors (52 women and 34 men, average age of 33 years) were all healthy and were geographically and ethnically matched to the patients.

Data concerning exposure to carcinogens in patients and controls were not available. Data concerning exposure to carcinogens in patients and controls were not available. Inclusion criteria for the PUD group included the following: age between 18 and 99 years; peptic ulcer disease; and *Helicobacter pylori* infection. Exclusion criteria included the following: age under 18 or over 99 years; treatment with non-steroidal anti-inflammatory drugs; and diagnosed cancer of the stomach or other organs. For the GC group, inclusion criteria included the following: age between 18 and 99 years; and diagnosis of stomach cancer. Exclusion criteria included the following: age below 18 or above 99 years; undiagnosed gastric cancer; and diagnosis of cancer of other organs. The investigation was carried out in accordance with the principles of the Declaration of Helsinki and was approved by the Ethics Committee of the Medical University of Lodz (RNN/195/13/KE). All subjects included in the study gave their informed consent.

### 2.2. Rapid Urease Test

In the group of peptic ulcer patients, *Helicobacter pylori* infection was diagnosed with the use of the rapid urease test (Institute of Food and Nutrition, Warsaw, Poland). The test is based on the fact that *H. pylori* synthesizes the enzyme urease, responsible for the conversion of urea to ammonium ions and carbon dioxide. In the case of a positive sample, the rapid urease test changes from yellow (negative) to red (positive).

### 2.3. DNA and RNA Isolation

DNA and RNA were isolated using a column-based method with chaotropic salts, in accordance with the following protocols: Genomic Mini, Blood Mini, and Total RNA Mini (A&A Biotechnology, Gdansk, Poland). The purity and concentration of DNA and RNA were assessed nanospectrophotometrically. Until analysis, DNA and RNA samples were stored at −20 °C and −80 °C, respectively.

### 2.4. Genotyping by Restriction Fragment Length Polymorphism (RFLP)

The polymerase chain reaction (PCR) was performed using the 2xPCR Super Master Mix (Biotool.com, San Francisco, CA, USA) in accordance with the manufacturer’s protocol. The PCR mixture consisted of the following: 5 μL of 2xPCR Super Master Mix, 0.5 mM of each forward and reverse primer, 50 ng of DNA template, and distilled water up to 20 μL. A negative control was included in every experiment. The PCR products were assessed by electrophoresis in 2% agarose gel. The primer sequence was taken from previous research of Yildiz et al. [5].

The products including the SNP at position G-248A were 438 bp in length. The amplified DNA fragments for SNP at position G-248A were digested by *AciI* (New England, BioLabs, Ipswich, MA, USA,) in 37 °C for 16 h. The digestion mixture consisted of 16 μL of PCR product, 2 μL of buffer, 0.2 μL of restriction enzyme, and 10 U/μL and 1.8 μL of distilled water. Genotypes were identified by digestion of PCR products followed by electrophoresis [5]. The products comprised one 352 bp band for genotype AA, two bands of 256 and 96 bp for genotype GG, and three bands of 352, 256, and 96 bp for genotype GA.

### 2.5. Expression Analysis by qPCR

In the first stage, all RNA samples were unified to an identical concentration of 0.05 µg/µL by adding appropriate volumes of distilled water. cDNA samples were obtained using a High-Capacity cDNA Reverse Transcription Kit (Applied Biosystems, Foster City, CA, USA) as per the manufacturer’s protocol. The reagent mixture for one sample contained the following: 2.0 µL 10  ×  RT Buffer, 2.0 µL oligo (dT) (0.5 µg/µL), 0.8 µL 25  ×  dNTP Mix (100 mM), 1.0 µL MultiScribe Reverse Transcriptase (50 U/µL), 1.0 µL RNase inhibitor, and 10 µL pre-prepared RNA. cDNA was stored at −20 °C until further analysis. *GAPDH* (glyceraldehyde-3-phosphate dehydrogenase) was used as a reference gene. The mRNA expression of the *BAX* and *GAPDH* genes was determined using real-time PCR (GAPDHF 5′-ACAGTTGCCATGTAGACC-3′; R 5′-TTGAGCACAGGGTACTTTA-3′; BAXF 5′AACTGGACAGTAACATGGAG-3′; R 5′-TTGCTGGCAAAGTAGAAAAG-3′).

Clinical samples were analyzed in triplicate using CFX Connect Real-Time PCR Detection System (BioRad, Hercules, CA, USA) in accordance with the iTaq Universal SYBR Green Supermix (BioRad, USA) protocol. The reaction mixture consisted of 5 µL RT PCR Mix, 0.35 μL reverse primer, 0.35 μL forward primer, 1 μL of cDNA, and 3.3 μL of distilled water. A negative control was included in every performed experiment. Melting temperature was determined to confirm the specificity of the obtained PCR product. After obtaining the Ct value for both the studied and reference genes, the relative expression levels were calculated using the ΔΔCt method [13].

### 2.6. Statistical Analysis

All statistical analyses were performed using STATISTICA 13 (StatSoft Inc., Tulsa, OK, USA, 2018). The Chi^2^ Pearson test was used to evaluate conformity between the observed and expected genotype frequencies according to the Hardy–Weinberg principle. To determine the significance of differences in genotype frequencies between the peptic ulcer patients group and the healthy individual group, Chi^2^ Pearson and Chi^2^ NW were used. These statistical tests were also applied to determine the significance of age, gender, infection by *H. pylori*, and severity of infection in the group of patients with peptic ulcers.

The groups and subgroups were relatively small. Therefore, Fisher’s exact test and the Chi^2^ NW were used to determine the significance of the differences in allele and genotype frequencies between the gastric cancer patients and healthy individuals, as well as the significance of age, gender, TNM, and degree of histological malignancy.

The U-Mann–Whitney test was used to determine the significance between R value and demographic, clinical, and pathological parameters in the peptic ulcer or gastric cancer groups. In contrast, the ANOVA test was used to compare the relationship between the SNP genotypes and *BAX* gene expression in the gastric cancer patients. Spearman’s rank correlation was used to evaluate the relationship between *BAX* and *NFKB2* gene expression. In all conducted tests, a *p* value of <0.05 was assumed as significant.

Where applicable, multiple testing correction was applied using the Benjamini–Hochberg method; the FDR value was set at 0.05. The adjusted *p* values are presented as q values [https://dreams.ucsc.edu/DreamSAT/multiple_comparisons.php; accessed on 27 March 2025].

### 2.7. In Silico Analysis

The *BAX* gene expression level was tested in gastric cancer tissue according to individual cancer stage, grade, and nodal methastasis status and also in comparison to normal tissue using TCGA-STAD (The Cancer Genome Atlas Stomach Adenocarcinoma) dataset through UALCAN’s (University of ALabama at Birmingham CANcer data analysis Portal; https://ualcan.path.uab.edu/analysis.html) online tool (accessed on 12 December 2023). A statistical comparison was performed using Student’s *t*-test with a *p*-value < 0.05 being significant [14,15].

Differential expression analysis of the Gene Expression Omnibus (GEO) microarray GSE264263 dataset (https://www.ncbi.nlm.nih.gov/geo/query/acc.cgi?acc=GSE264263; accessed on 25 March 2025) was performed with the GEO2R online tool. This set comprises Affymetrix Human Gene 1.0 ST Array data of gastric adenocarcinoma cell line (AGS) infected and uninfected with *Helicobacter pylori* (3 biological replicates per treatment and control groups). We assumed padj < 0.05 to be statistically significant. For visualization of differential analysis results, we used EnhancedVolcano package in RStudio 2024.12.0.

The correlation between the expression of *BAX* gene and *NFKB2* and *TP53* genes in the gastric cancer TCGA dataset was assessed using the Spearman correlation using TIMER2 (Tumor IMmune Estimation Resource 2.0; https://db3.cistrome.org/browser/) database (accessed on 12 December 2023 and 28 March 2025). A *p*-value < 0.001 was considered significant [16].

The Kaplan–Meier plotter (https://kmplot.com/analysis/index.php?p=background) was used to perform overall survival analysis depending on BAX gene expression in the whole group of patients with gastric cancer and also depending on stage, grade, nodal, and distant metastasis (accessed on 12 January 2024). The “Auto select best cut-off “ option was utilized to divide the BAX gene’s low- and high-expression groups, and *p*-value was calculated using the log-rank test. Significance was considered to be *p* < 0.05 [17].

The RNAStructure Software Package (version 6.4; rna.urmc.rochester.edu; accessed on 10 January 2024) was used for DNA secondary structure prediction and change in free energy ΔG calculation. All parameters were set to default values.

Protein–protein interactions were assessed using the STRING web tool (https://cn.string-db.org/; accessed on 30 March 2025). It enables the assessment of direct–physical and indirect–functional associations. In the current analysis, 20 interactions with a minimum confidence at level 0.9 were predicted. The k-means clustering of the generated PPI networks for the BAX protein was performed with a present of three clusters (marked as red, green, and blue) [18].

## 3. Results

### 3.1. Genotyping of the BAX Gene

All DNA samples from 183 peptic ulcer patients, 13 gastric cancer patients, and 86 healthy individuals were successfully analyzed (see Table 1).

An in silico comparison of the free energy change (ΔG) of the most stable structure between wild-type or allelic variant was used to test the influence of the studied SNP on DNA structure. The substitution of nucleotide G with A at position −248 led to a decrease in free energy from −462.1 to −465.3. It should be recognized that, although the presence of the A variant may have a minor effect on the thermodynamic stability of DNA, it could still influence the transcription process by altering the secondary structure or modulating interactions with regulatory proteins. However, further in vitro research should be performed to confirm these findings.

#### 3.1.1. Genotyping of SNP G-248A in the Group of Peptic Ulcer Patients

Genotype frequencies for the SNP were compared between the group of patients with peptic ulcer and the healthy individuals. No statistically significant differences were found (*p* = 0.2079; q = 0.62352). This may indicate that the studied SNP is not associated with the risk of developing peptic ulcer disease (Table 1).

Based on rapid urease test results, the peptic ulcer patients were divided into two subgroups, namely those infected with *Helicobacter pylori* and those that were not. The subgroup of patients infected with *H. pylori* was then subdivided into two subgroups of patients: one with mild bacterial infection (+) and the other with moderate (++) or severe infection (+++). No statistically significant differences were found for either subgroup (Table 2). To sum up, the tested polymorphism is not related to the risk of *H. pylori* infection or its severity.

#### 3.1.2. Genotyping of SNP G-248A in the Group of Gastric Cancer Patients

For the assessment of the risk of gastric cancer development, the frequency of G248A SNP genotypes was compared between the gastric cancer and healthy individuals groups (see Table 1). It was found that AA homozygous occurred in the group of gastric cancer patients, but not in the group of healthy individuals (*p* < 0.001; q = 0.006). This result may indicate a potential relationship between the genotype AA of the studied polymorphism and the risk of developing gastric cancer.

No significant difference was found between the prevalence of the SNP G-248A genotype and TNM classification or grade of histological malignancy in the group of patients with gastric cancer (Supplemental Table S1).

Our findings relate to gastric cancer and may be influenced by the small number of patients in the study group; therefore, our results should be carefully interpreted and treated more as exploratory data. All obtained findings should be verified in a larger group of gastric mucosa biopsies taken from patients with gastric cancer. After conducting experimental studies, we attempted to perform in silico analysis for this polymorphism using available data from GEO database concerning gastric cancer. Unfortunately, we were unable to find a dataset that would be similar to our study group.

### 3.2. BAX Gene Expression Level

The relative expression level of the *BAX* gene in samples from patients, 36 with peptic ulcer, 13 with gastric cancer, and 6 tissues collected beyond the margin of cancer tissue and macroscopically considered as healthy, were successfully analyzed.

#### 3.2.1. Expression of *BAX* mRNA in Peptic Ulcer Patients

Relative expression of the *BAX* gene was highly variable among the patients with peptic ulcer disease (see Table 3). The group was subdivided into those infected with *Helicobacter pylori* and those who were not, based on the results of the rapid urea test. The uninfected group was found to demonstrate over six times higher mRNA expression of *BAX* gene (median: 2.3924) than the infected group (median: 0.3862); this difference was significant (*p* = 0.0207; q = 0.1449). After using the Benjamini–Hochberg correction method, the analysis lost its statistical significance, which indicates the need for further verification using a larger group of patients. All results are summarized in Table 3.

#### 3.2.2. Expression of *BAX* mRNA in Gastric Cancer Tissue and In Silico Analysis of TCGA- STAD Data and GSE264263 Data

**Figure 1:**
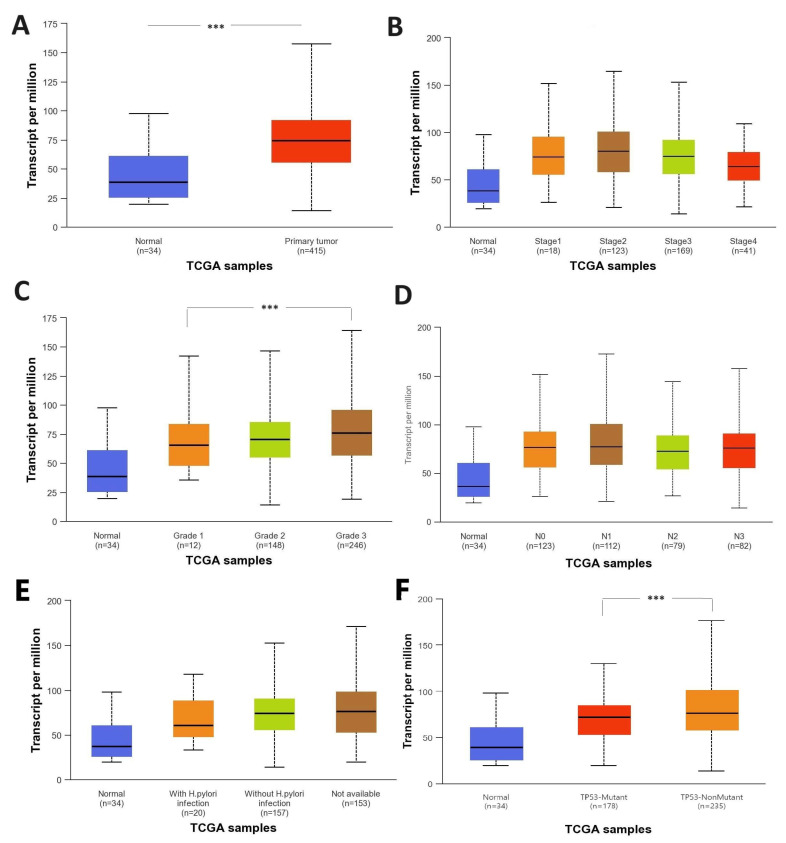
Box-whisker plots showing the expression of the BAX gene in normal and gastric cancer tissue (**A**), according to clinical stage (**B**), histological malignancy grade (**C**), nodal metastasis status of cancer (**D**), Helicobacter pylori infection status (**E**), and TP53 mutation status obtained by UALCAN database (**F**) (results are presented as median, lower and upper quartiles, and minimum and maximum. n-number of samples; Grade 1—well differentiated (low grade); Grade 2—moderately differentiated (intermediate grade); Grade 3—poorly differentiated (high grade); N0—metastases into regional lymph node; N1—metastases in one to three axillary lymph nodes; N2—metastases in four to nine axillary lymph nodes; N3—metastases in ten or more axillary lymph nodes; *** *p* < 0.001; accessed on 12 December 2023).

**Figure 2:**
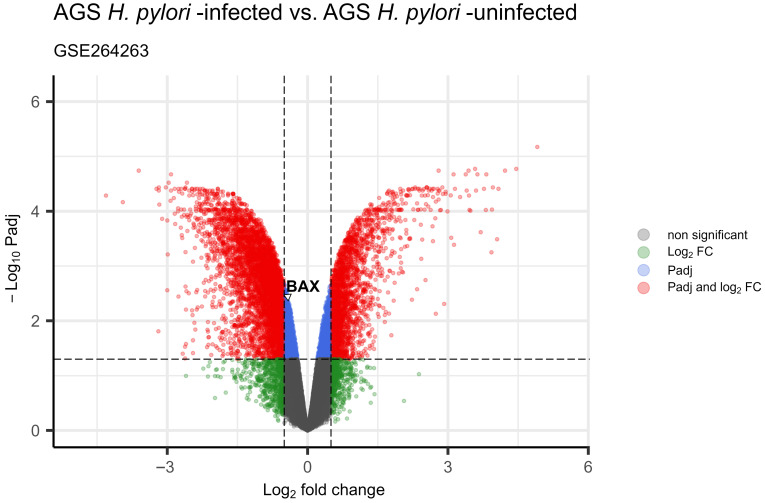
Volcano plot for differential expression analysis of microarray GSE264263 dataset (threshold |log2FC| > 0.5 and padj < 0.05); *BAX* expression level is significantly decreased in *H. pylori*-infected gastric adenocarcinoma AGS cell line as compared to uninfected control (log2FC = −0.45; padj = 0.0037).

In the group of patients with gastric cancer, *BAX* gene expression level was variable. No statistically significant correlations were found according to TNM (*p* = 0.1375) or degree of histological malignancy (*p* = 0.2840) (see Table 3). Unfortunately, analysis taking into account H. pylori infection status was not possible in case of GC group (wet analysis) because this data were unavailable. In silico studies using the TCGA-STAD (The Cancer Genome Atlas Stomach Adenocarcinoma) data form the UALCAN database showed upregulated expression of the *BAX* gene in gastric cancer tissue compared to normal tissue (*p* < 0.001) (Figure 1A). Moreover, it was shown that the expression of this gene was significantly higher in patients with a poorly differentiated (grade 3) than in patients with well-differentiated (grade 1) cancer tissue (*p* < 0.001) (Figure 1C). However, *BAX* gene expression was not associated with the clinical advancement of gastric cancer or lymph node metastasis (Figure 1B,D). Despite the fact that, similarly to our peptic ulcer patients, *BAX* gene expression was higher among patients not infected than those infected with *Helicobacter pylori*, this was not shown to be a statistically significant relationship (*p* = 0.4349) (Figure 1E). In line with this, differential expression analysis of the microarray GSE264263 dataset revealed a lower *BAX* expression level in the gastric adenocarcinoma AGS cell line infected with *Helicobacter pylori* when compared with uninfected AGS counterparts; in that case, the difference was statistically significant (log2FC = −0.45; padj = 0.0037) (Figure 2). Then, analysis of the *BAX* gene showed a difference in relation to the *TP53* status, where higher levels of expression were observed in mutant *TP53* patients compared to wild-type TP53 (*p* < 0.001, Figure 1F).

#### 3.2.3. Comparison of *BAX* Gene Expression Between Peptic Ulcer Patients, Gastric Cancer Patients, and Morphologically Normal Tissue Samples

**Figure 3:**
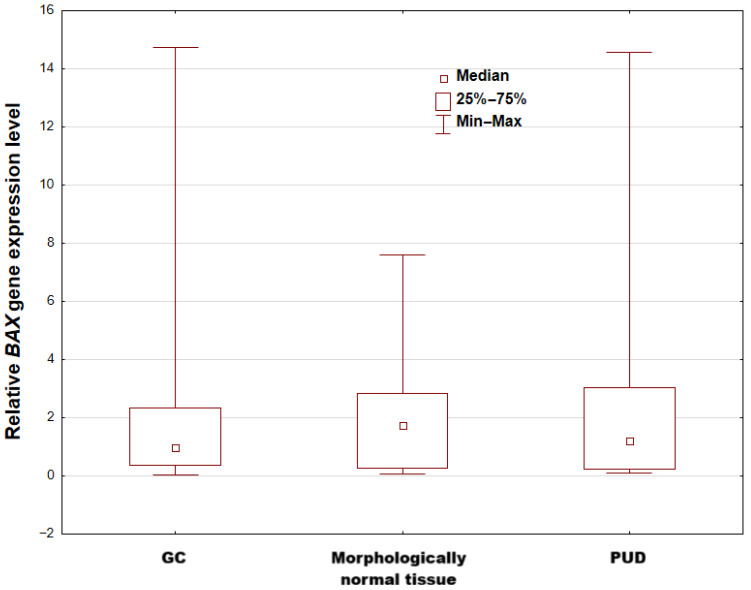
Relative *BAX* gene expression level in two investigated groups and control cohort (results are presented as median, lower and upper quartiles, and minimum and maximum. PUD—biopsies of gastric mucosa collected from patients with peptic ulcer; GC—gastric tissue collected from patients with gastric cancer; morphologically normal tissue—macroscopically normal gastric tissue collected beyond the margin of cancer tissue from patients with gastric cancer).

No significant differences in median *BAX* mRNA expression were found between peptic ulcer cases (median 1.2000) and macroscopically normal tissue (median 1.4382) (*p* = 0.7875; q = 0.8264), nor between the gastric cancer group (median: 1.4241) and morphologically normal tissue (median 1.4382); (*p* = 0.8264; q = 0.8264), nor between peptic ulcer patients (median 1.2000) and gastric cancer patients (median 1.4241); (*p* = 0.6263; q = 0.8264) (see Figure 3). However, the obtained results may be a consequence of the size of the individual groups of patients.

### 3.3. Assessment of the Correlation Between SNP G-248A Genotype and BAX Expression

The relative *BAX* gene expression with regard to SNP G-248A individual genotypes was estimated in the gastric cancer group. No significant differences were found (*p* = 0.1686; q = 0.3934). All data are summarized in Supplemental Table S2.

### 3.4. Interrelation Between BAX Gene Expression Level and Other Target Genes

Considering that the function of the *BAX* gene can potentially be influenced by other signaling pathways, its expression was compared with previous data regarding *NFKB2* gene expression obtained from the same group [19]. No correlation was found between the two genes in either the PUD (r2 = 0.0000; *p* = 0.9970) or GC (r2 = 0.0070; *p* = 0.7852) patients. However, in the case of gastric cancer, in silico studies using the TIMER database showed a positive correlation between *NFKB2* and *BAX* gene expression (r = 0.341; *p* < 0.001) (Supplemental Figure S1).

Furthermore, as indicated by the analysis performed using the STRING web tool, the BAX protein can interact with many other proteins. The network showed, with a confidence score of at least 0.9, that the top 20 most functional partners for BAX were as follows: MCL1, BCL2L1, BCL2, BID, BCL2L11, BAK1,TP53, CYCS, VDAC1, XRCC6, TMBIM6, BBC3, SIRT1, PRKN, PPIF, BCL2L2, AIFM1, PRKACA, PRKACB, and PRKACG. Within the generated PPI network, three clusters of interaction proteins for the studied BAX were identified: red, green, and blue. 

**Figure 4:**
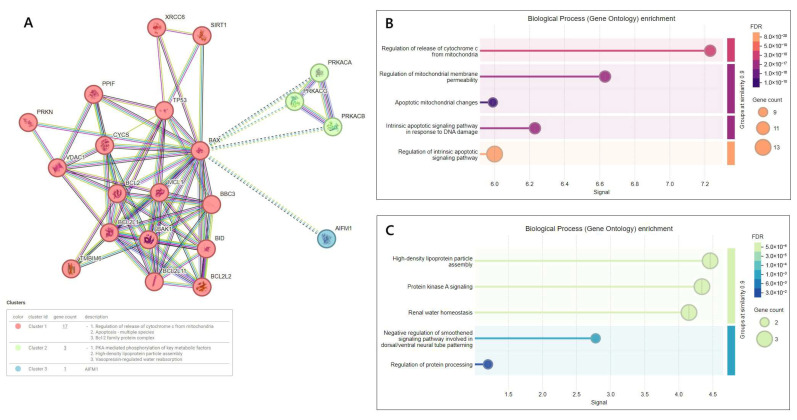
Cluster analysis for BAX by using STRING database (the identified clusters are marked as red, green, and blue) (**A**); and functional enrichment of cluster red (**B**); and cluster green and blue (**C**) (*Y*-axis—biological processes, transcribed to a set of genes/proteins: the higher they are on the axis, the more specific the given biological process was for them; *X*-axis—statistical significance of the biological process: the lower it is, the more statistically significant the dataset is; Gene count—point size: the number of genes/proteins involved in a given process; FDR—enrichment strength: the darker the color, the stronger the enrichment; accessed on 30 March 2025).

Using the TIMER database showed a positive correlation between TP53 and BAX gene expression (r = 0.261; *p* < 0.001) (Supplemental Figure S2), which is considered a transcriptional activator of the BAX (Figure 4). In our research, the correlation between them was not assessed in clinical samples.

### 3.5. Prognostic Value of BAX Gene Expression Level in Gastric Cancer

**Figure 5:**
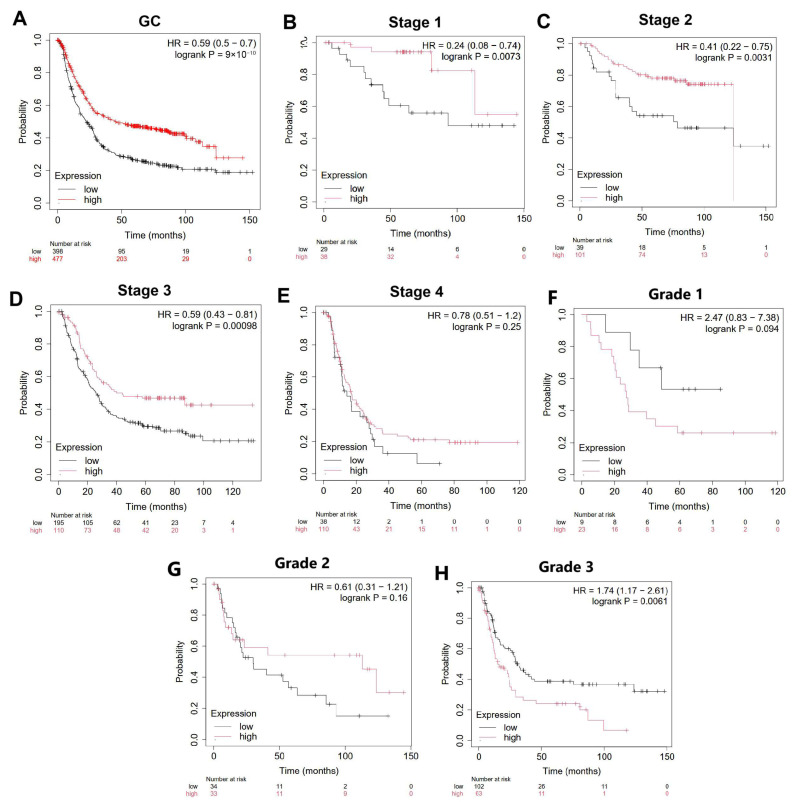
The correlation of *BAX* gene expression with overall survival in a whole group of patients at (**A**) stage 1, (**B**) stage 2, (**C**) stage 3, (**D**) stage 4, (**E**) grade 1, (**F**) grade 2, (**G**) and grade 3 (**H**) with gastric cancer (GC) using Kaplan–Meier plotter database (red—high expression; black—low expression; HR—hazard ratio; accessed on 12 January 2024).

To determine if *BAX* gene expression is a prognostic factor in gastric cancer, the Kaplan–Meier plotter database was used. We found that upregulated expression of *BAX* mRNA was correlated with a better prognosis in terms of overall survival (OS) (*p* < 0.05; Figure 5A.). The median of OS was longer in subgroups of patients with higher expression of the *BAX* gene. This may be related to the proapoptotic effect of *BAX* on cancer cells. After that, the influence of *BAX* gene expression on OS was analyzed depending on clinicopathological features such as grade, stage, nodal metastasis, and distant metastasis. Increased *BAX* expression was associated with longer overall survival in patients with clinical stage 1, stage 2, and stage 3 (*p* < 0.05; Figure 5B–E). Similarly, upregulated *BAX* expression was associated with longer OS in patients with and without the presence of metastases in lymph node (Supplemental Figure S3) and distant organs (Supplemental Figure S4). The opposite results were obtained in patients with poorly differentiated tissue (grade 3), where higher *BAX* levels were associated with shorter overall survival (*p* < 0.05; Figure 5F–H).

It was not possible to verify this thesis in our wet research due to the lack of information on the survival time of the patients recruited in this study.

## 4. Discussion

Apoptosis is a physiological process that helps to maintain the balance between the formation of new cells and the death of old and damaged ones through the equilibrium of anti-apoptotic and pro-apoptotic factors [20]. The protein encoded by the *BAX* gene promotes apoptosis via the internal pathway [7]. Research suggests that polymorphisms/mutations in the *BAX* gene may be associated with changes in mRNA and protein expression, which may translate into an increased risk of developing diseases, including cancer [5].

To determine whether the SNP G-248A is a potential risk factor for the development of peptic ulcer diseases (PUD), the present study compared the results of *BAX* genotyping between peptic ulcer patients and healthy individuals. While the AA genotype was more common among peptic ulcer patients than healthy individuals, the difference was not significant, which may indicate that its presence does not seem to increase the risk of peptic ulcer disease. Unfortunately, as we did not find any similar research on PUD, it was impossible to compare our results with other studies in this field. However, as PUD has an inflammatory background, our findings may be compared with those of other inflammatory diseases. For example, patients with osteomyelitis, a disease often associated with bacterial infection, were also found to be more likely to demonstrate the SNP G-248A polymorphism AA genotype; however, this difference was not significant, nor was it associated with the presence of any microorganism that could underlie the development of osteomyelitis [21]. Our findings indicate that the AA genotype is not associated with the presence of infection of *H. pylori* or its severity.

In contrast, previous studies suggest that the SNP may contribute to the development of neoplastic diseases. It is likely that the polymorphism at position G-248A is associated with decreased *BAX* promoter activity and, consequently, a decreased amount of encoded protein, inhibition of apoptosis, and promotion of tumor growth. Indeed, the *BAX* protein has been found to be less prevalent in esophageal squamous cell carcinoma tissue with the AA G-248A SNP compared to GG [11]; hence, the present study compares the *BAX* G-248A genotypes between patients with gastric cancer and healthy individuals. Interestingly, it was found that homozygous AA occurred in the group of gastric cancer patients, but not in healthy individuals, suggesting that its presence was a risk factor. It is possible that the presence of the AA genotype is associated with the deletion of a binding site for transcription factors, which may lead to a reduction in the expression of this gene and thus the inhibition of apoptosis [20]. However, this SNP is probably not connected with cancer progression, as no statistically significant association was found between particular variants and TNM or histological grade. Mirmajidi et al. do not note any correlation between the presence of the AA variant of G-248A and increased risk of gastric cancer development and progression [20]. Yildiz et al. and Bhatt et al. also found no significant difference between the *BAX* SNP genotype and clinical stage or grade in breast cancer [5,22]. The study by Olbromski et al. also did not indicate any association of this *BAX* gene polymorphism with the risk of developing ovarian cancer [23]. However, similar results to ours were obtained by Javid et al., who note a relationship between the AA genotype and increased risk of non-small-cell lung cancer (NSCLC) development [24], and Chen et al., where the AA genotype occurred more frequently in squamous cell carcinoma of the head and neck [25]. The AA genotype was also significantly more common in patients with nasopharyngeal carcinoma and in patients with papillary thyroid cancer [26,27]. Despite the fact that our study did not reveal any association between SNP and gastric cancer progression, Javid et al. showed that, in NSCLC, the AA genotype was more frequent in patients with more advanced, poorly differentiated cancer with metastases. The AA genotype was identified as a negative prognostic factor for survival in NSCLC [24]. However, it is worth remembering that cancer progression is complex process, involving different molecular mechanisms, and *BAX* alone may not be responsible for it. Many other proteins participate in the apoptosis pathway, such as BCL-2 or p53; perhaps simultaneous changes in the expression of these proteins or their interactions with *BAX* are more decisive for disease progression than *BAX* alone. Epigenetic changes or the tumor microenvironment may also have an impact, especially in the context of *H. pylori* infection. Furthermore, the role of *H. pylori* in inducing oxidative stress, DNA damage, chronic inflammation, and cell apoptosis, as well as its impact on the tumor microenvironment, was shown. On the other hand, abnormal methylation of genes, for example, IGF2, SLC16A2, SOX11, P2RX7, and MYOD1, were identified in *H. pylori* infection and significantly correlated with gastric cancer and its clinicopathological features [28].

Our present findings indicate that the three types of investigated tissues demonstrated similar mRNA expression levels of the *BAX* gene, with a slightly lower level in gastric mucosa specimens from peptic ulcer disease. Similarly, Iimura et al. reported that *BAX* expression was reduced in active ulcerative colitis [29]; however, Bartchewsky et al. found higher *BAX* mRNA expression in chronic gastritis than in gastric cancer and higher *BAX* gene expression in ulcers with *Helicobacter pylori* infection [30]. Also, in the study by Hai-Feng et al., an increase in *BAX* protein level was demonstrated in metaplasia and dysplasia of gastric tissue with current *H. pylori* infection [31]. In addition, in the study by Zhang et al., higher *BAX* levels were noted in gastric cancer in the presence of *H. pylori* [32], which may suggest that the bacterial infection increases *BAX* expression levels and promotes apoptosis; however, our present findings indicate that *BAX* expression was more than seven times higher in the subgroup of PUD patients without *H. pylori* infection. These differences may arise from the small size of the studied groups, but also from the fact that both Bartchewsky and Zhang detected *H. pylori* that were cagA-positive and the specific vacA genotype. It is worth remembering that *H. pylori* cagA can influence apoptosis, not only through *BAX*, but also through various signaling pathways such as JNK, PI3K/AKT, and NF-kB [33]. In the present study, it is not known whether infection was present in patients with gastric cancer, and nor can the status of the cagA and vacA gene in the case of *Helicobacter pylori* infection in both investigated groups of patients with GC and PUD [30,32]. Thus, it is possible that *BAX* gene expression is not directly related to the development of peptic ulcer disease or gastric cancer. However, due to the limited size of the study groups, these findings should be interpreted with caution. This is particularly relevant for the case of gastric cancer, where our in silico studies showed that higher levels of *BAX* gene expression occurred in cancer tissue, and increased mRNA was a positive prognostic factor.

*BAX* function can be stimulated by proteins from various signaling pathways, including the NF-kB pathway, which modulate the activation of numerous genes involved in inflammation, proliferation, and apoptosis. Studies show that *Helicobacter pylori*, the major factor responsible for PUD and GC development, activates the non-canonical NF-kB pathway connected with the *NFKB2* gene. Ohmae et al.’s study proposed that *H. pylori* may attenuate apoptosis via the alternative NF-kB2 pathway, and inhibition of this physiological process may be associated with an increased risk of cancer development; hence, the present study compared *BAX* expression with our previous data regarding *NFKB2* in the same PUD and GC cohorts; however, no such interrelation was found. Nevertheless, the existence of a potential link between these two genes should be further investigated, as other studies indicate that another pathogen, such as the *Epstein–Barr Virus* (EBV), may inhibit pro-apoptotic *BAX* activity by binding *NFkB* to BAX domains [20,34,35,36,37]. In addition, the influence of *TP53*, which is considered to be an activator of *BAX* transcription and thus associated with the mitochondrial apoptosis pathway, may be important. Rajabi-Moghaddam et al. indicated that the G-248A *BAX* SNP alone was not associated with the risk of developing squamous cell carcinoma of the head and neck (HNSCC). However, simultaneous presence of a *TP53* heterozygote and an AA homozygote for the studied *BAX* SNP was associated with an increased risk of developing this cancer [38]. Similarly, in the study by Mrózek et al., the combination of mutated *TP53* and *BAX* (this time frameshift mutation) was associated with more aggressive cancer, poor prognosis, and faster GC recurrence [39]. In pituitary adenocarcinomas, reduced expression of *BAX* and *VDAC1* genes were associated with resistance to apoptosis and thus promoted tumor development. Another study shows that *TRIM17*, through interaction with *BAX*, which results in its proteasomal degradation, led to the inhibition of apoptosis and excessive cell proliferation in the gastric cancer cell lines AGS and HGC-27 [40,41]. It seems that *BAX* may become a very important risk of development marker in diseases, including cancers. On the other hand, it may also be associated with response to treatment. For example, reduced *BAX* expression was associated with resistance to cisplatin treatment in cancer cell lines, and in ESCA it was associated with shortened OS time in patients treated with cisplatin [24]. However, in some studies, *BAX* overexpression was an indication of the need for more intensive or alternative therapy [42]. Kazemi et al. also showed that treatment of AGS gastric cancer cells with high concentrations of saffron led to increased *BAX* expression and promoted apoptosis [43]. In conclusion, the results of this exploratory study indicate that the presence of the AA SNP G-248A *BAX* genotype may be a significant risk factor for the development of gastric cancer, and its overexpression may be a positive prognostic marker in GC.

## 5. Conclusions

The findings of our study indicate a potential association between the AA genotype of the G-248A polymorphism in the *BAX* gene and an increased risk for gastric cancer, whereas this genetic variant does not seem to affect peptic ulcer disease susceptibility. Contrarily, *BAX* gene mRNA expression levels do not exhibit a direct correlation with the incidence of either gastric cancer or peptic ulcer disease. These observations, especially regarding gastric cancer, are based on a limited sample size, necessitating cautious interpretation, as they are preliminary.

The study faced significant limitations due to the constrained number of samples within each group and the minimal volume of available gastric cancer tissue and mucosal specimens. This limitation impacted our capacity for conducting multiple nucleic acid extractions from individual samples, as detailed in the Section 2. Additionally, the relatively rare incidence of gastric cancer, coupled with its frequent late-stage diagnosis in Poland, complicates the collection of a substantial group within a brief period. The samples utilized for gastric cancer and adjacent normal tissue were derived from archival sources.

Despite these constraints, the insights gained from this research lay a solid groundwork for future investigations into the *BAX* gene and its involvement in the molecular pathways underlying peptic ulcer disease and gastric cancer. Future studies with larger and more diverse groups are critical for validating our preliminary findings and deepening our understanding of the genetic factors contributing to investigated diseases development. In future studies, we plan not only to expand the study groups, but also to perform in vitro studies and functional analyses, including the effect of the G-248A polymorphism on the transcription of the *BAX* gene.

## Figures and Tables

**Table 1:** Genotype distribution of SNP G-248A *BAX* in peptic ulcer patients, gastric cancer patients, and healthy individuals.

*BAX* G-248A	Peptic Ulcer Patients N = 183	Healthy Individuals N = 86	*p*	q
GG	142 (77.6%)	63 (73.3%)	0.2079	0.62352
GA	37 (20.2%)	23 (26.7%)		
AA	4 (2.2%)	0 (0.0%)		
HWE ^$^ (*p* values)	0.9053	0.3300		
	**Gastric Cancer Patients** **(N = 13)**	**Healthy Individuals** **(N = 86)**		
GG	1 (7.7%)	63 (73.3%)	<0.001	0.006
GA	10 (76.9%)	23 (26.7%)		
AA	2 (15.4%)	0 (0.0%)		
HWE ^$^ (p values)	0.2545	0.3300		

$—Hardy–Weinberg equilibrium.

**Table 2:** The comparison of G-248A *BAX* gene genotype frequencies between peptic ulcer patients with *H. pylori* infection and those without.

BAX G-248A	Peptic Ulcer Patients N = 183			
	Infected N = 90	Uninfected N = 93	*p*	q
GG	67 (74.5%)	75 (80.6%)	0.4393	0.6235
GA	20 (22.2%)	17 (18.3%)		
AA	3 (3.3%)	1 (1.1%)		
***H. pylori* Infection Severity** **(N = 90) assessed at**				
	**(+)** **N = 47**	**(++) or (+++)** **N = 43**		
GG	34 (37.8%)	33 (36.7%)	0.8306	0.8306
GA	11 (12.2%)	9 (10.0%)		
AA	2 (2.2%)	1 (1.1%)		

**Table 3:** Relative *BAX* mRNA level in the group of peptic ulcer and gastric cancer patients and morphologically normal tissue.

	Relative *BAX* mRNA Level	N	Median	Min.	Max.	Lower Quartile	Upper Quartile	*p*	q
PEPTIC ULCER	All cases	36	1.2000	0.0949	14.5829	0.2483	3.0201		
	Women	21	0.4784	0.0949	14.5829	0.2237	2.9887	0.5421	0.6324
	Men	15	1.6543	0.0967	9.6657	0.4806	3.0515		
	Uninfected with *H. pylori*	26	2.3924	0.1014	14.5829	0.3305	3.6205	0.0207	0.1449
	Infected with *H. pylori*	10	0.3862	0.0949	1.9402	0.1851	1.0043		
	Severity of infection assessed at (+)	6	0.2384	0.0949	1.9402	0.0967	0.4806	0.2410	0.3976
	Severity of infection assessed at (++) or (+++)	4	0.8443	0.2021	1.8313	0.4432	1.4178		
GASTRIC CANCER	All cases	13	1.4241	0.0477	14.7230	0.3950	3.9724		
	Women	5	1.4241	0.3950	8.2249	0.9794	1.4743	1.0000	1.0000
	Men	8	1.3608	0.0477	14.7230	0.3029	7.4503		
	TNM Tis or I	6	2.8329	0.9794	10.9283	1.0281	8.2249	0.1375	0.3934
	TNM II or III	7	0.3950	0.0477	14.7230	0.2570	1.4743		
	G1	6	2.8329	0.3487	10.9283	0.9794	8.2249	0.2840	0.3976
	G2 or G3	7	1.0281	0.0477	14.7230	0.2570	1.4743		
MORPHOLOGICALLY NORMAL TISSUE	All cases	6	1.4382	0.0677	5.1860	0.1523	2.8375		

## Data Availability

The original contributions presented in this study are included in the article/Supplementary Material. Further inquiries can be directed to the corresponding authors. On the other hand the datasets used for in silico analysis in this study are available publicly, links to the archives: https://ualcan.path.uab.edu/analysis.html (accessed on 12 December 2023); http://timer.cistrome.org/ (accessed on 12 December 2023), https://kmplot.com/analysis/ (accessed on 12 January 2024), rna.urmc.rochester.edu (accessed on 10 January 2024), https://www.ncbi.nlm.nih.gov/geo/query/acc.cgi?acc=GSE264263; accessed on 25 March 2025); (https://dreams.ucsc.edu/DreamSAT/multiple_comparisons.php; accessed on 27 March 2025), https://cqsclinical.app.vumc.org/ps/; accessed on 27 March 2025).

## Supplementary Materials

The following supporting information can be downloaded at https://www.mdpi.com/article/10.3390/cimb47121005/s1.

## Abbreviations

The following abbreviations are used in this manuscript:

AGS	gastric adenocarcinoma cell line
BAX	B-cell lymphoma 2-associated X protein
EBV	Epstein–Barr Virus
G1	Grade 1—well differentiated (low grade)
G2	Grade 2—moderately differentiated (intermediate grade)
G3	Grade 3—poorly differentiated (high grade)
GAPDH	glyceraldehyde-3-phosphate dehydrogenase
GC	gastric cancer
GEO	Gene Expression Omnibus
HNSCC	Squamous cell carcinoma of the head and neck
HWE	Hardy–Weinberg equilibrium
N0	metastases into regional lymph node
N1	metastases in one to three axillary lymph nodes
N2	metastases in four to nine axillary lymph nodes
N3	metastases in ten or more axillary lymph nodes
NSAIDs	non-steroidal anti-inflammatory drugs
OS	overall survival
PPI	protein–protein interaction
PUD	Peptic ulcer disease
SNP	single nucleotide polymorphism
TCGA-STAD	The Cancer Genome Atlas Stomach Adenocarcinoma
TIMER2	Tumor IMmune Estimation Resource 2.0
UALCAN	University of ALabama at Birmingham CANcer data analysis Portal
