# Supplementary material for: In Silico and Wet Analysis of BAX Gene G-248A Polymorphism and mRNA Expression in Peptic Ulcer Disease and Gastric Cancer
Source: Curr Issues Mol Biol. 2025 Nov 29;47(12):1005. doi: 10.3390/cimb47121005 (PMC12731365; doi:10.3390/cimb47121005)

Supplementary Material

Supplemental Table S1. A comparison of G248A *BAX* gene genotype frequencies between TNM and degree of histological malignancy subgroups of gastric cancer patients.

		BAX G-248A			**p**	**q**
		**GG**	**GA**	**AA**		
**TNM**	Tumor *in situ* or I	0 (0.0 %)	5 (38.45 %)	1 (7.7 %)	*0.5196*	*0.62352*
	II or III	1 (7.7 %)	5 (38.45 %)	1 (7.7 %)		
**Degree of histological malignancy of the tumor**	G1	0 (0.0 %)	5 (38.45 %)	1 (7.7 %)	*0.5196*	*0.62352*
	G2 or G3	1 (7.7 %)	5 (38.45 %)	1 (7.7 %)		

Supplemental Table S2. *BAX* gene expression level with regard to SNP G-248A genotypes.

Relative *BAX* mRNA level	N	Median	Min.	Max.	Lower quartile	Upper quartile	p	q
GG	1	0.0477	N/A	N/A	N/A	N/A	***0.1686***	***0.3934***
GA	10	1.2261	0.2570	14.7230	0.3950	3.9724		
AA	2	6.2013	1.4743	10.9283	1.4743	10.9283		

Supplemental Table S3. TNM stage and grade of histological malignancy in the group of gastric cancer patients.

		Number of cases
**TNM**	*Tumor in situ*	1
	I	5
	II	5
	III	2
**Grading**	G1	6
	G2	5
	G3	2

Supplemental Figure S1. The correlation between *NFKB2* and *BAX* gene expression obtained using TIMER database (access: 12.12. 2023).

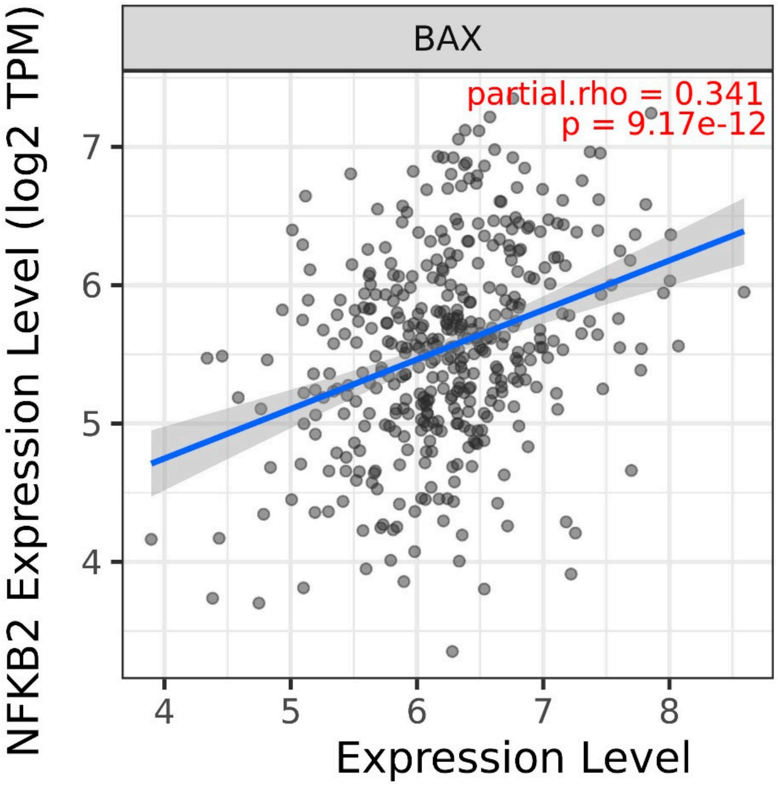

Supplementary Figure S2. The correlation between *TP53* and *BAX* gene expression obtained using TIMER database (access: 27.03.2025).

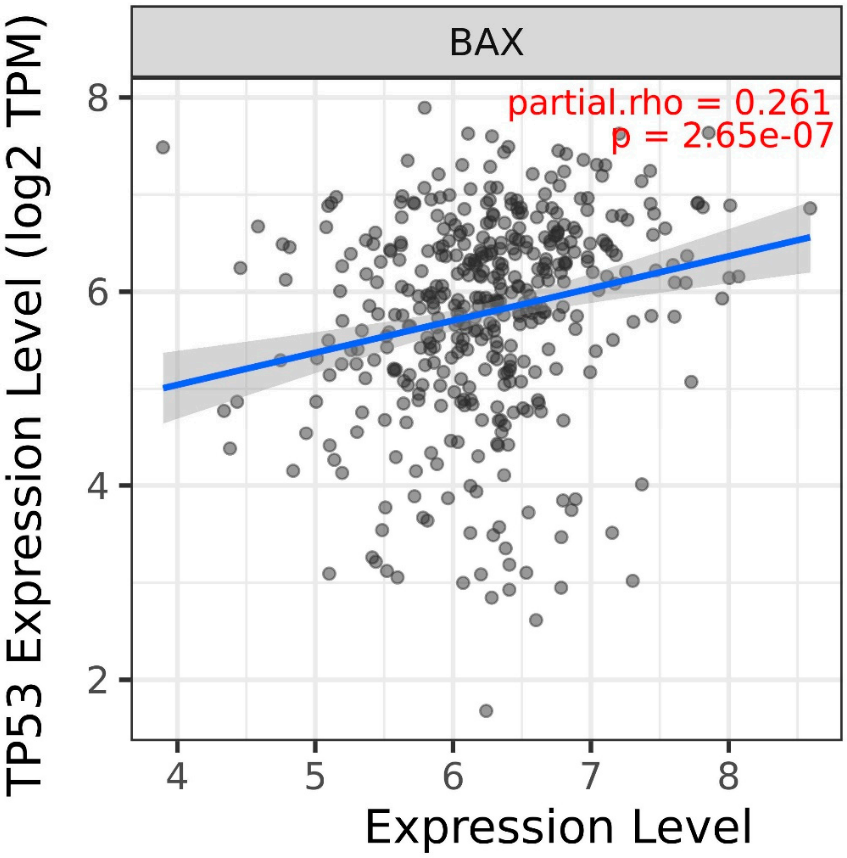

Supplementary Figure S3. The correlation of *BAX* gene expression with overall survival depending on absent (A) or present (B) metastasis into nodal in gastric cancer (GC) using Kaplan-Meier plotter database ((red - high expression; black - low expression; HR- hazard ratio access: 12.01.2024).

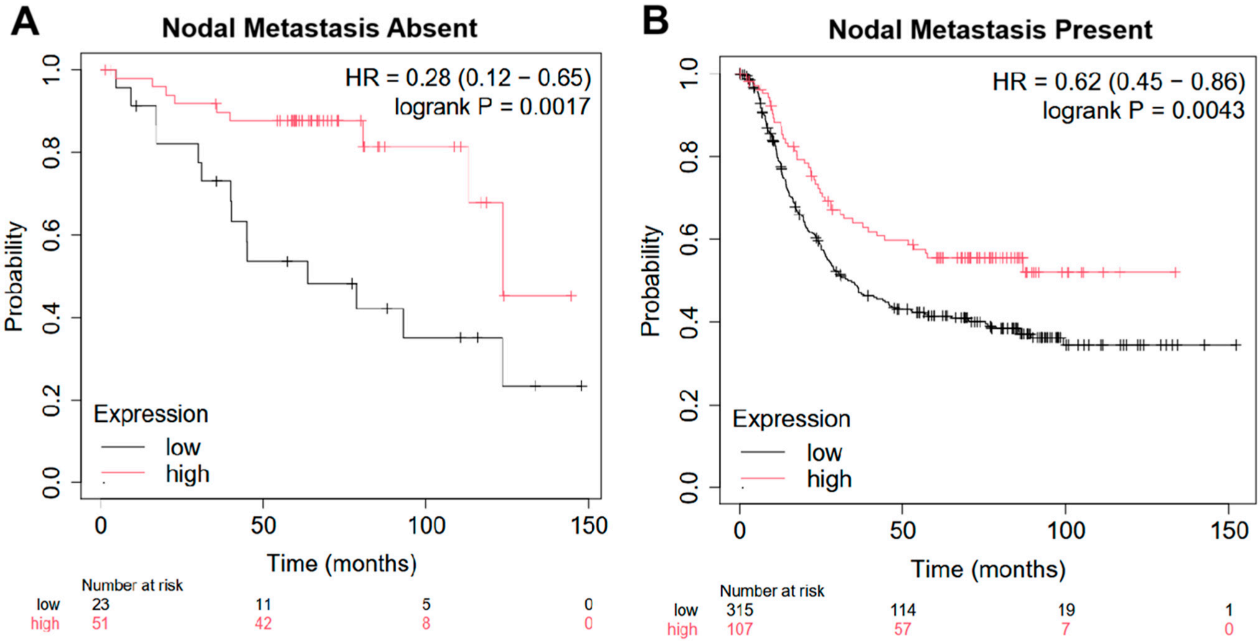

Supplementary Figure S4. The correlation of *BAX* gene expression with overall survival depending on absent (A) or present (B) of metastasis into distant organs in gastric cancer (GC) using Kaplan-Meier plotter database (red - high expression; black - low expression; HR- hazard ratio access: 12.01.2024).

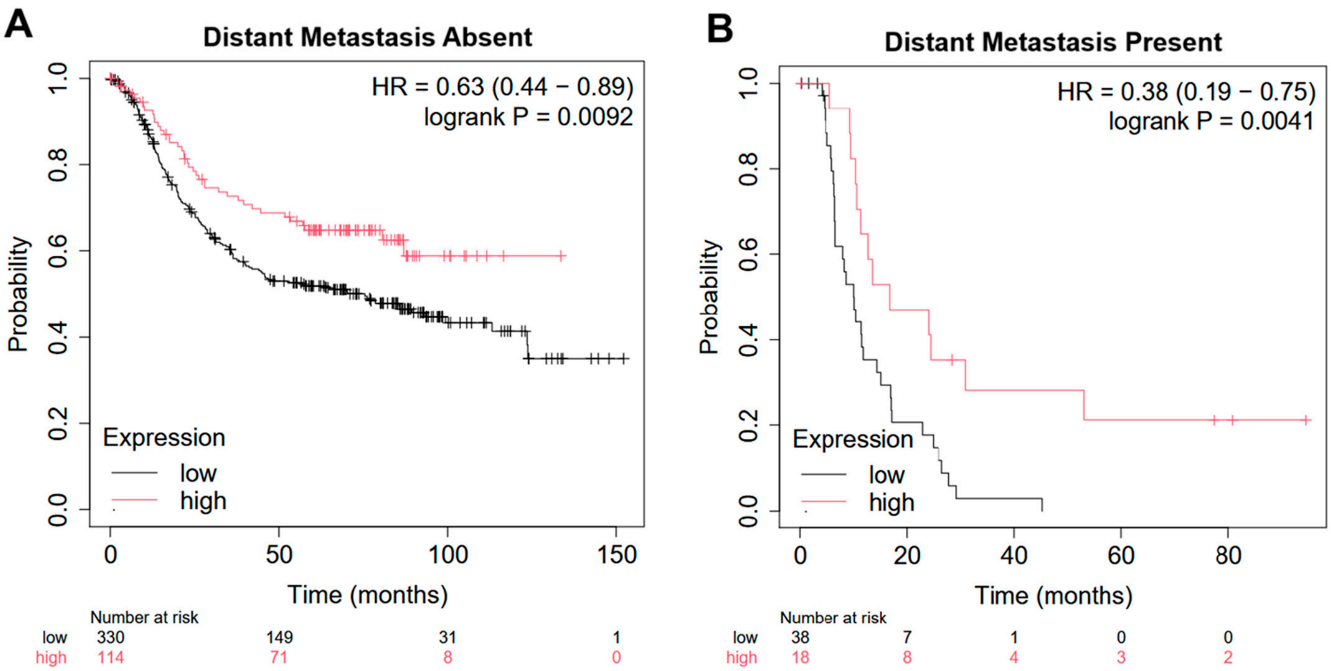

Supplement: Supplementary file 1 [file cimb-47-01005-s001.zip › cimb-4001983-supplementary.pdf]
